# The Effects of Spinal Manipulation Added to Exercise on Pain and Quality of Life in Patients with Thoracic Spinal Pain: A Randomized Controlled Trial

**DOI:** 10.1155/2023/7537335

**Published:** 2023-04-27

**Authors:** Muhammad Sharif Waqas, Hossein Karimi, Ashfaq Ahmad, Shazia Rafiq, Naveed Anwar, Sidrah Liaqat

**Affiliations:** ^1^The University of Lahore, Pakistan; ^2^Istanbul Gelisim University, Turkey; ^3^Allama Iqbal Medical College Lahore, Pakistan; ^4^Nur International University, Lahore, Pakistan; ^5^Mayo Hospital Lahore, Pakistan

## Abstract

**Background:**

There are not enough reliable studies available in physiotherapy to determine the effects of spinal manipulative therapy added to exercise on thoracic spinal pain and quality of life.

**Objective:**

To investigate the effects of spinal manipulation on pain and quality of life in subjects with thoracic spinal pain. *Study Design*. It was an open-label “randomized controlled trial.” *Study Settings*. Department of Physiotherapy, Services Hospital, Lahore, Pakistan. *Participants*. There were one hundred subjects with an age group between 18 and 60 years fulfilling the inclusion criteria. These subjects were divided equally into two groups; an experimental and a control group.

**Methods:**

In the experimental group (*n* = 50), thoracic spinal manipulation was applied along with thoracic muscle strengthening exercises. In the control group (*n* = 50) thoracic muscle exercises alone were given. Pain was measured by visual analogue scale (VAS) and quality of life with SF-36. Measurements were taken at baseline, immediately after session, after 8th session, and later as follow-ups at 12 weeks. Repeated measure ANOVA and independent sample *T*-test were used for within and between-group comparisons.

**Results:**

Mean age of subjects in control group was 38.56 ± 12.44 and in experimental group was 36.02 ± 11.32. Both groups demonstrated significant improvement in VAS score, and all domains of SF 36 but between-group comparison showed greater improvement in VAS of the experimental group compared to the baseline (*P* < 0.05), but between-group comparison of 8th session to follow-up has shown that effects of exercise persist while health-related quality of life in spinal manipulation group was significantly reduced after discontinuation of treatment. After the 8th session, spinal manipulation group showed notable results in terms of pain (mean diff 1.14 (0.62, 1.65) 95% CI and all aspects of SF 36 (*P* value <0.05). However, after week 12 of follow-up, no significant difference (*P* value >0.05) was observed among the study groups for pain and quality of life.

**Conclusion:**

Spinal manipulation added to thoracic exercise was more effective than thoracic exercise alone for improving pain and quality of life at the end of 8th session of care. However, the inclusion of spinal manipulation was not found effective at the 12-week follow-up. This trial is registered with IRCT20190327043125N1.

## 1. Introduction

Spinal pain is considered the leading cause of disability in individuals worldwide. But thoracic spinal pain, which might be but not exclusively related to other spinal pathologies, is an underexplored region. Epidemiological data on thoracic pain in the general population is also limited as compared to other spinal regions. It can be as disabling as pain in other regions and therefore needs attention [1, 2].

An array of risk factors is identified for the development of thoracic pain such as repetitive tasks, prolonged static posture, spinal pathologies, and psychological stresses. These factors may result in altered biomechanics of discs and soft tissues [1].

Many techniques are suggested in the literature for the treatment of spinal pain, commonly including massage, exercises, mobilization, and electrical modalities [3]. But there is a lack of supporting evidences of interventions that target thoracic spine as the primary source of the symptom. One approach to conservative treatment includes manual manipulation of thoracic spine. It is hands-on clinical approach that uses high velocity low amplitude thrust and is adopted by many professionals. Its clinical effectiveness in other areas such as cervical, lumbar, and shoulder has also been shown [4].

It is suggested that manual therapy shows improvement in symptoms by exerting biomechanical and neurophysiological effects [5]. The neurophysiological effect can stimulate sympathetic nervous system, this in turn can increase skin conductance, respiratory rate and heart rate, suggesting sympathetic excitatory effect [6, 7]. It also produces hypoalgesia by affecting pain processing centers, endocrine responses, and increasing pressure pain threshold [8].

In the medical community, pain is considered a challenging problem. As it is a complex pathophysiological process, it can have an impact on social and psychological well-being of a person. Persistent spinal pain also causes an increase in absentees from work and increase in treatment cost. Researches are needed to correctly diagnose and treat the cause of thoracic spinal pain as the literature does not comprehensively elucidate this area [9]. It was hypothesized that spinal manipulation with thoracic spinal exercises is more effective than thoracic spinal exercise alone on pain and quality of life in patients with thoracic spinal pain.

## 2. Method

### 2.1. Design

A parallel group randomized controlled trial was conducted. Participants, willing to participate in this study, were included from Physiotherapy Department Services Hospital Lahore, Pakistan. Random allocation into two groups was made by a computerized generated randomization table. SNOSE method was used for the concealment of allocation. In SNOSE, an independent researcher with no clinical involvement made envelops. Except for therapist who was giving treatment, all other staff including assessors and participants were blinded to the given treatment. Subjects were not aware of treatment provided in the other arm of the study, and the persons administering the questionnaires about pain and quality of life were blinded to the results at the other time points.

### 2.2. Participants, Therapists, and Centers

For this study, a total of 127 subjects were assessed, out of which 100 participants including both male and female, ages between 16 and 60 years, who were having nonspecific thoracic spine pain, in the spinal area T1 to T12, mobility deficit in thoracic spinal range of motion, and having pain with compression on the thoracic spine, were included in the study. All other participants who had a contraindication to manual therapy, including osteoporotic, thoracic spinal fractures, spinal infection, metastatic disorders, spondyloarthropathy, and disc herniation, and had a history of referring pain to the thoracic spine due to visceral conditions were excluded from the study. All the participants gave written informed consent.

### 2.3. Intervention

The experimental group received thoracic spinal manipulation consisting of a high velocity and low amplitude thrust in the prone position. Manipulation was applied to hypomobile spinal segments with joint-play restriction identified by palpation with posterior-anterior and transverse pressure. This technique was considered successful when an auditory or palpable release was perceived by a therapist. Manipulation maneuver was administered by a physiotherapist with high thrust and low amplitude force, with more than 10 years of clinical experience. The control group only received thoracic spinal muscle exercises with three sets of 10 repetitions and a rest period of 1 minute between the intervals. The position of subjects for exercises was sitting and prone lying, and medium resistance TheraBand was used. Scapular retraction exercises were performed in a sitting position with elbows bent at 90 degrees, and the subjects pulled the TheraBand backwards to move shoulder blades towards each other [10]. In prone lying, second exercise was performed, arms flexed and extending the thoracic spine [11, 12]. Third exercise with TheraBand around the arms was performed for thoracic rotation, in sitting. Fourth exercise was performed either in a sitting or prone lying position, with trunk side flexion. Both groups received ergonomic advice for maintaining correct anatomical posture.

If the subject improved in the follow-up session so that there was no pain, and the motion was normal, then, only the exercises were performed in the follow-up visits. During the treatment, patients were not allowed to take care from other clinics or health care providers for this musculoskeletal condition.

Participants were treated 2 times per week for four weeks, and a total of 8 sessions were given. Measurements for pain were taken at baseline, after 1st session of 4 weeks and after the 8th session. Measurements for SF 36 were taken at baseline and after the 8th session. Patients were followed up at the 12th week after randomization (3rd month) to check the long-term effects.

### 2.4. Outcome Measures

#### 2.4.1. Visual Analogue Scale (VAS)

It is a 10 cm line with one end indicating no pain and other maximum possible self-perceived pain. The subject marks a line between the two-end reflecting his/her level of pain which is then measured as a distance from the first end. VAS is considered a valid and reliable measure of self-perceived musculoskeletal pain in previous studies [13].

#### 2.4.2. Short Form 36 (SF-36) Questionnaire

SF 36 is a valid a reliable measure that gauges the quality of life. It consists of 36 questions related to the self-perceived quality of life which are further subgrouped as physical functioning, role limitation due to physical health, role limitation due to emotional problems, energy/fatigue, emotional well-being, social functioning, pain, and general health. The score of each category ranged from 0 to 100 with higher scores indicating better quality of life [13].

### 2.5. Sample Size

The sample size was determined with mean VAS score in group 1 (*μ*_1_) = 7.6, SD in group 1 (*σ*_1_) = 8.6 [14], mean in group 2 (*μ*_2_) = 12.2, and SD in group 2 (*σ*_2_) = 9.2 [14] from a previous pilot study, ratio (*r*) = 1.00, alpha (*α*) = 0.05, Z (0.975) = 1.959964, beta (*β*) = 0.200, Z (0.800) = 0.841621. The sample size in each group was 45. 50 patients in each group were taken by assuming 10% drop out rate.

### 2.6. Data Analysis

**Figure 1:**
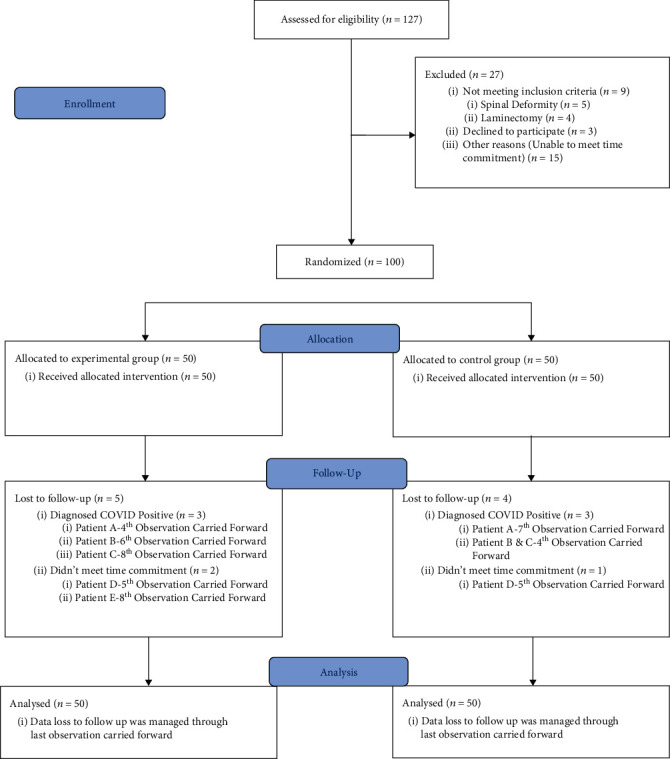
CONSORT flow sheet diagram.

Data was analyzed on SPSS version 23. Descriptive analysis (mean, variance, and standard deviation) was performed for quantitative data. Frequencies and percentages were calculated for categorical and nominal data of gender. Data was analyzed for normality by applying the Kolmogorov–Smirnov test, which showed that data was normally distributed (*p* value >0.05). An intention-to-treat analysis was performed, and all subjects were included in the analysis in the group to which they were randomized. Independent sample *t*-test was used for between-group comparisons. Repeated measure ANOVA with a Greenhouse-Geisser correction was used for within-group analysis. *p* value ≤0.05 was considered as significant. Last observation carried forward (LOCF) was used to handle the missing data due to loss of follow-up. This technique replaces a participant's missing values after dropout with the last available measurement and assumes that the participant's responses would have been stable from the point of dropout to trial completion, rather than declining or improving further Figure 1.

## 3. Results

Demographics and baseline measurements for the VAS score and 8 domains of SF 36 were comparable (Table 1). There was a statistically significant change with each group for VAS score and all domains of SF 36 (*p* value <0.001) (Table 2). The control and experimental group differed significantly for VAS score and all domains of SF 36 till the 8th session of treatment (Table 3). However, no significant difference was reported across both groups at follow-up on 12 weeks. (Table 4).

## 4. Discussion

This is the first trial evaluating the effect of adding spinal manipulation to thoracic exercise for the treatment of thoracic back pain. The purpose of this study was to gather evidence for the effects of thoracic spinal manipulation on pain and health-related quality of life in subjects with thoracic spinal pain. By analyzing the results, it was shown that there was a significant change within the difference between groups with a reduction in pain and an improvement in quality of life.

The results of the present study show that there is a significant difference between groups in the reduction of pain. A group that was given thoracic spine manipulation showed improvement at the end of the 8th session on the VAS scale (Table 3). This is in concomitant with other studies as different theories can explain the hypoalgesia effect of manual therapy by sympathetic nervous system activation [6]. However, neurophysiological effects of spinal manipulation that produce analgesia are shown to be short termed [15, 16]. As shown in this study, scores on the pain scale, immediately after the session and after the 8^th^ session, showed significant improvement while measurements after a longer term follow-up of two months did not show any significant difference between the two groups. These results of reduction in pain are similar to spinal manual therapy applied to other areas such as cervical and lumbar region [6, 17].

Pain is regarded as psychophysiological phenomenon, as it has psychological and physical components. In the present study, this was measured by SF 36 in terms of quality of life of subjects. Results showed significant improvement in spinal manipulation group as compared to exercise group. The improvement in this spinal manipulation group may be due to an effective decrease in pain after only 1 session. The results also persisted for longer duration as measured after 12 weeks, when treatment was discontinued.

This is also evident in other spinal areas that manipulation reduces pain and improves function [18, 19]. Both physical and mental components of SF 36 showed improvement within both groups, but between-group comparison has shown that spinal manipulation was more effective in improving health-related quality of life immediately after treatment and effects were lost after the discontinuation of manipulation treatment but persists in exercise group which is persistent with previous literature [17]. There was a statistically significant difference present between the 8th session and follow-up at 12th week (*p* < 0.05 for all domains) within experimental group, showing that health status quality of life was significantly altered, and effects of thoracic manipulation was not persistent for up to 2 months after the discontinuation of manipulation.

In treating a patient, holistic- and patient-centered approach is required and acknowledging the fact that psychological, environmental, nutritional, and emotional factors may have an impact on physical and social aspects of individuals [20].

## 5. Limitations

Some potential limitations of this study are that data was collected only from a single setting, and follow-up was just up to 12 weeks, so in the future, researches should be conducted based on several treatment sessions with even longer follow-ups, and multicenter randomized controlled trials are needed. A force applied in this technique was not assessed, so dosage remains a matter of concern for further studies. Chronicity of pain should have been identified as acute, and chronic pain might have different effects on treatment outcome.

## 6. Conclusion

Spinal manipulation in addition to thoracic exercise was more effective than thoracic exercise alone for improving thoracic pain and quality of life at the end of the 8th session of care. The advantage of including spinal manipulation was not found at the 12-week follow-up because improvement abated in the manipulation group, whereas improvement in the control group tended to be stable. This may be due to a short-term benefit of spinal manipulation in the treatment regimen or regression to the mean.

## Figures and Tables

**Table 1:** Baseline comparison of the mean (SD) of the VAS score and the 8 domains of SF 36 between the control and experimental group.

Variables	Control (*n* = 50)	Experimental (*n* = 50)
Gender (% males)	70 (*n* = 35)	68 (*n* = 34)
Age (years)	38.56 ± 12.44	36.02 ± 11.32
VAS score	6.58 (1.41)	6.50 (1.55)
Physical functioning	25.12 (20.22)	27.10 (20.12)
Role limitation due to physical health	30.60 (19.07)	33.70 (26.02)
Role limitation due to emotional problems	35.32 (21.74)	38.15 (23.58)
Energy/fatigue	25.10 (16.97)	25.69 (18.33)
Emotional well-being	26.58 (22.21)	25.04 (18.42)
Social functioning	29.92 (23.06)	29.40 (17.85)
Pain	21.89 (19.14)	22.54 (16.57)
General health	27.44 (16.77)	27.00 (19.40)

**Table 2:** Comparison of the mean (SD) of the VAS score and the 8 domains of SF 36 within experimental and control groups.

Outcome	Treatment groups	Baseline (*n* = 50)	Immediate after 1st session (*n* = 50)	After 8th session (*n* = 50)	Follow-up at 12th week (*n* = 50)	*p* value
VAS score	Control	6.58 (1.41)	6.44 (1.41)	3.40 (1.48)	2.74 (1. 06)	<0.001
	Experimental	6.50 (1.55)	4. 58 (1.72)	2.26 (1.08)	2.46 (1.23)	<0.001

Physical functioning	Control	25.12 (20.22)	xxxxxx	55.46 (22.35)	56.36 (21. 07)	<0.001
	Experimental	27.10 (20.12)	xxxxxx	64.70 (16.94)	62.90 (18.29)	<0.001

Role limitation due to physical health	Control	30.60 (19.07)	xxxxxx	51.80 (26.14)	49.80 (26.61)	<0.001
	Experimental	33.70 (26.02)	xxxxxx	62.60 (22.57)	54.90 (22.91)	<0.001

Role limitation due to emotional problems	Control	35.32 (21.74)	xxxxxx	53.10 (27.71)	52.79 (27.45)	<0.001
	Experimental	38.15 (23.58)	xxxxxx	66.11 (24.30)	57.90 (20.09)	<0.001

Energy/fatigue	Control	25.10 (16.97)	xxxxxx	47.40 (17.93)	45.60 (16.67)	<0.001
	Experimental	25.69 (18.33)	xxxxxx	57.10 (24.49)	49.90 (19.96)	<0.001

Emotional well-being	Control	26.58 (22.21)	xxxxxx	45.92 (19.41)	44.64 (19.57)	<0.001
	Experimental	25.04 (18.42)	xxxxxx	58.30 (20.07)	51.98 (22.35)	<0.001

Social functioning	Control	29.92 (23.06)	xxxxxx	60.86 (15.63)	57.75 (15.27)	<0.001
	Experimental	29.40 (17.85)	xxxxxx	69.49 (14.34)	57.35 (15.06)	<0.001

Pain	Control	21.89 (19.14)	xxxxxx	60.32 (17.29)	61.60 (12.97)	<0.001
	Experimental	22.54 (16.57)	xxxxxx	70.32 (18.17)	64.36 (16.00)	<0.001

General health	Control	27.44 (16.77)	xxxxxx	60.50 (15. 05)	59.80 (15.18)	<0.001
	Experimental	27.00 (19.40)	xxxxxx	67.8 (14.43)	62.90 (16.93)	<0.001

**Table 3:** Across the group differences for the VAS score and the 8 domains of SF 36 after the 8th session.

Outcome	Control (*n* = 50)	Experimental (*n* = 50)	Mean difference (95% CI)	*p*-value
VAS score	3.40 (1.48)	2.26 (1.08)	1.14 (0.62, 1.65)	<0.001
Physical functioning	55.46 (22.35)	64.70 (16.94)	9.24 (-17.11, -1.36)	0.02
Role limitation due to physical health	51.80 (26.14)	62.60 (22.57)	10.80 (-20.49, -1.10)	0. 02
Role limitation due to emotional problems	53.10 (27.71)	66.11 (24.30)	13. 01 (-23.35, -2.66)	0.01
Energy/fatigue	47.40 (17.93)	57.10 (24.49)	9.70 (-18.22, -1.17)	0.02
Emotional well-being	45.92 (19.41)	58.30 (20. 07)	12.38 (-20.21, 4.54)	<0.001
Social functioning	60.86 (15.63)	69.49 (14.34)	8.63 (-14.58, -2.67)	<0.001
Pain	60.32 (17.29)	70.32 (18.17)	10. 00 (-17. 04, -2.95)	<0.001
General health	60.50 (15. 05)	67.8 (14.43)	7.30 (-13.15, -1.44)	0.01

**Table 4:** Across the group differences for the VAS score and the 8 domains of SF 36 after the 12th-week follow-up.

Outcome	Control (*n* = 50)	Experimental (*n* = 50)	Mean difference (95% CI)	*p* value
VAS score	2.74 (1. 06)	2.46 (1.23)	0.28 (-0.17, 0.73)	0.22
Physical functioning	56.36 (21. 07)	62.90 (18.29)	6.54 (14.37, 1.29)	0.10
Role limitation due to physical health	49.80 (26.61)	54.90 (22.91)	5.10 (14.95, 4.75)	0.30
Role limitation due to emotional problems	52.79 (27.45)	57.90 (20. 09)	5.10 (14.65, 4.43)	0.29
Energy/fatigue	45.60 (16.67)	49.90 (19.96)	4.30 (11.60, 3.00)	0.24
Emotional well being	44.64 (19.57)	51.98 (22.35)	7.34 (15.67, 0.29)	0.08
Social functioning	57.75 (15.27)	57.35 (15. 06)	0.40 (-5.62, 6.42)	0.89
Pain	61.60 (12.97)	64.36 (16. 00)	2.76 (-8.54, 3.02)	0.34
General health	59.80 (15.18)	62.90 (16.93)	3.10 (-9.48, 3.28)	0.33

## Data Availability

The data will be available on request.
